# Csf1 Signaling Regulates Maintenance of Resident Macrophages and Bone Formation in the Mouse Cochlea

**DOI:** 10.3389/fneur.2019.01244

**Published:** 2019-11-21

**Authors:** Takayuki Okano, Ippei Kishimoto

**Affiliations:** Department of Otolaryngology, Head and Neck Surgery, Graduate School of Medicine, Kyoto University, Kyoto, Japan

**Keywords:** resident macrophages, inner ear, osteopetrosis, bone remodeling, hearing loss

## Abstract

In the mammalian cochlea, resident macrophages settle in the spiral ligament, spiral ganglion, and stria vascularis, even at the steady state. Resident macrophages in the cochlea are believed to maintain homeostasis in the inner ear and become active, as part of the front line defense, following inner ear damage. However, the exact roles of cochlear resident macrophages require further clarification. Colony stimulating factor-1 (Csf1) signaling regulates survival, proliferation, and differentiation of resident macrophages and appears to be essential for resident macrophages in the inner ear. To examine the roles of Csf1 signaling in auditory function, we examined the ossicles and inner ear of homozygous *Csf1* mutant (*Csf1*^*op*/*op*^) mice. The ossicles including the incus and stapes of *Csf1*^*op*/*op*^ mice macroscopically demonstrated bone thickening, and the otic capsules of the inner ear were also thick and opaque. Histological analyses demonstrated that the otic capsules in *Csf1*^*op*/*op*^ mice were thickened and showed spongy bone degeneration. Measurements of the auditory brainstem response revealed significant elevation of thresholds in 4-week old *Csf1*^*op*/*op*^ mice compared with *wild-type* littermates, indicating that *Csf1*^*op*/*op*^ mice demonstrate hearing loss due to, at least in part, deformity of the ossicles and bone capsule of the inner ear. Furthermore, *Csf1*^*op*/*op*^ mice are deficient in the number of resident macrophages in the spiral ligament and stria vascularis, but not in the spiral ganglion. These data provide evidence that Csf1 signaling is important not only for bone formation in the inner ear, but also for the maintenance of resident macrophages in the spiral ligament and stria vascularis in the adult mouse cochlea.

## Introduction

The inner ear was once believed to be “immune-privileged,” because the concentration of IgG in the perilymph is as low as in the cerebrospinal fluid and there is no lymphatic drainage or lymphoid tissue. However, a new era of inner ear immunology began following the discovery of intimate contacts between lymphocytes and macrophages in the endolymphatic sac of guinea pigs (1). In addition, previous studies have demonstrated the presence of immune-competent cells in specific parts of the inner ear such as the spiral ganglion and the spiral ligament, which are referred to as resident macrophages in the cochlea (2, 3).

Tissue resident macrophages are distributed in virtually all organs and tissues in the body and provide crucial innate immune defense and tissue-specific functions in the regulation and maintenance of organ homeostasis (4). Recent studies revealed considerable heterogeneity of macrophages with respect to both development and metabolism and have provided convincing evidence that diverse lineages of tissue resident macrophages determine their responses to immune stimulation and environmental stressors (5). Under pathological conditions, macrophages acquire inflammatory effector functions, but they can also develop regulatory properties essential for tissue protection and repair. Moreover, macrophages recruited during inflammation are often functionally distinct from tissue resident macrophages (6). In the cochlea of adult mice, resident macrophages expressing Iba1 settle in both the spiral ligament and the spiral ganglion even in the steady state. Once inflammation caused by acoustic overstimulation or bacterial infection occurs in the cochlea, circulating monocytes migrate, and gather at wounded sites (3, 7–9). Cochlear resident macrophages are thought to be important for the immune system of the inner ear, including maintenance of the local environment and repair of damaged tissue or cells. However, many aspects regarding the functions of cochlear macrophages remain to be elucidated.

Colony stimulating factor-1 (Csf1, also known as MCSF or Csfm) signaling regulates survival, proliferation, and differentiation of resident macrophages (10). Mice homozygous for the naturally occurring *Csf1*^*op*^ (op: osteopetrosis) gene mutation lack *Csf1*, due to a spontaneous frameshift mutation, and have a reduced macrophage population in various organs (11). Due to a severe deficiency of osteoclasts that leads to a severe restriction in bone remodeling, *Csf1*^*op*/*op*^ mice possess extensive skeletal deformities and a lower body weight in addition to a lower life-span and very poor breeding performance (12).

Thus, *Csf1*^*op*/*op*^ mice offer the opportunity to understand the function of macrophages in the bony capsule (otic capsule) and organs surrounding the auditory sensory epithelium, such as the spiral ganglion and spiral ligament in the mouse cochlea. We herein examine and report the phenotypes in the inner ear of *Csf1*^*op*/*op*^ mice and further clarify the roles of Csf1 signaling in the mouse inner ear.

## Materials and Methods

### Animals

Mutant *Csf1*^*op*/*op*^ mice were bred in our laboratory from breeding pairs of B6C3F1-a/a, *Csf1*^*op*/+^ mice obtained from Dr. Toshio Suda, Keio University, Japan. *Csf1*^*op*/+^ mice carry a spontaneous mutation of the *Csf1* gene, a single nucleotide (thymidine) insertion 262 bp downstream from the initiation codon. This results in a frameshift and the generation of a stop codon 21 bp downstream of the insertion. Genotyping using tail snip was performed with polymerase chain reaction (PCR) with a primer set of Forward 1 for wild type (*WT*) (ATCCTGTTTGCTACCTAAAGAAGGCCATTT), Forward 2 for *Csf1*^*op*^ mutation (TCCTGTTTGCTACCTAAAGAAGGCCCATTT), and Reverse (CTTGTTCTGCTCCTCATAGTCCTTGGTGAA), followed by digestion with BstX-I. Mice were maintained in a pathogen-free microisolator environment in the Institute of Laboratory Animals, Kyoto University Graduate School of Medicine. Due to their lack of teeth, *Csf1*^*op*/*op*^ pups were started on a powdered diet formula when 3 weeks old. All animal procedures were performed in accordance with the NIH Guide for the Care and Use of Laboratory Animals and were approved by the Animal Research Committee of Kyoto University Graduate School of Medicine (No. 170510, MedKyo18117).

### Auditory Brainstem Responses and Noise Exposure

The auditory brainstem response (ABR) was measured under general anesthesia, through intraperitoneal injection of midazolam (10 mg/kg; Astellas Pharma Inc., Tokyo, Japan) and xylazine (10 mg/kg; Bayer DVM, Leverkusen, Germany). ABR waveforms were recorded using sound stimuli of tone bursts at 8, 16, and 32 kHz for 12.8 ms, at a sampling rate of 40 kHz, using 50–5,000 Hz band-pass filter settings, and were averaged from 1,000 stimuli. Stimulus intensity was decremented in 5-dB steps from high to subthreshold levels. The auditory threshold was defined as the lowest stimulus intensity that evoked a recognizable ABR wave pattern. All ABRs of experimental animals (*n* = 4, each genotype) were recorded at the age of 4 weeks and male and female mice were analyzed together.

To assess their susceptibility to acoustic overstimulation, animals were exposed to 8 kHz octave band noise at 120 dB sound pressure level (SPL) for 1 h in a ventilated sound exposure chamber while having free access to food and water. The sound levels were monitored and calibrated at multiple locations within the sound chamber to ensure uniformity of the stimulus. ABR measurements were performed pre-exposure and 2 h and 7 days after noise exposure.

### Histological Assessments

Under general anesthesia with midazolam and xylazine, animals were perfused intracardially with ice-cooled phosphate-buffered saline (PBS) followed by 4% paraformaldehyde in phosphate buffer. The temporal bones were collected and immersed in the same fixative for 4 h at 4°C. Samples were decalcified with 10% EDTA in PBS and cryoprotected with 30% sucrose. Specimens were prepared as cryostat sections (10 μm in thickness). Midmodiolar sections were obtained for histological analyses. Histological assessments of the otic capsule were performed with hematoxylin-eosin staining. To quantify the thickness of otic capsule, the vertical thickness of the otic capsule was measured in the basal turn where the basement membrane of cochlea is attached. In addition, the cross-sectional area of Rosenthal's canal and the width of habenula perforata were also measured in the basal turn (*n* = 4 for each genotype).

For immunohistochemical assessment of cochlear resident macrophages, cryostat sections were immersed in blocking solution containing 10% goat serum for 30 min and incubated with a primary antibody at 4°C overnight. Cochlear macrophages were labeled by immunostaining for ionized calcium binding adapter molecule 1 (Iba1), which is specific for microglia/macrophages (3). The primary antibody used in this study was rabbit anti-Iba1 (1:1,000; Wako Pure Chemicals, Osaka, Japan). Visualization of primary antibodies was performed using secondary antibodies conjugated with Alexa Fluor 555 (1:500; Molecular Probes, Eugene, OR). Nuclei were counterstained with 4′,6-diamidino,2-phenylindole dihydrochloride (DAPI, 1 μg/ml in PBS; Molecular Probes). Negative controls lacked primary antibody labeling. Specimens were viewed with a Nikon Eclipse E600 fluorescence microscope (Nikon, Tokyo, Japan).

Images were processed using Adobe Photoshop CS6 and Adobe Illustrator CS6 (Adobe Systems, San Jose, CA, USA). Image manipulations were limited to adjustments to brightness, hue, and saturation.

### Quantification of Cochlear Macrophages

For the quantification of Iba1-positive cells, four sections were selected randomly from the 12 most midmodiolar sections for each *WT* or *Csf1*^*op*/*op*^ animal. All cochlear macrophages, defined by coexpression of Iba1 and DAPI within the cochlea, were counted by two double-blinded examiners.

To investigate alterations in the number of Iba1-positive cells in the cochlea, the density of Iba1-positive cells in the spiral ganglion (SG, Rosenthal's canal), spiral ligament (SL, region occupied by type I-V fibrocytes), or stria vascularis (SV) was calculated by a modified method as described previously for evaluating the density of SG neurons (13). All Iba1-positive DAPI-stained cells within the SG, SL, or SV from the midbasal portion of the cochlea were counted. The SG, SL, and SV profiles were then traced under a bright field image to generate the area of SG with Image J (http://www.nist.gov/lispix/imlab/prelim/dnld.html). The density of Iba1-positive cells in SG, SL, or SV was expressed as the number of cells per 10,000 μm^2^.

### Statistical Analysis

Statistical analyses were performed by using one-way analysis of variance (ANOVA) followed by the Tukey-Kramer test for the analysis of ABR thresholds. An unpaired *t*-test was used in other statistical analyses. Data were expressed as the mean ± standard error. Differences with *p*-values below 0.05 were considered statistically significant.

## Results

### Excessive Bone Formation Is Pronounced in the Middle and Inner ear of *Csf1^*op*/*op*^* Mice

**Figure 1:**
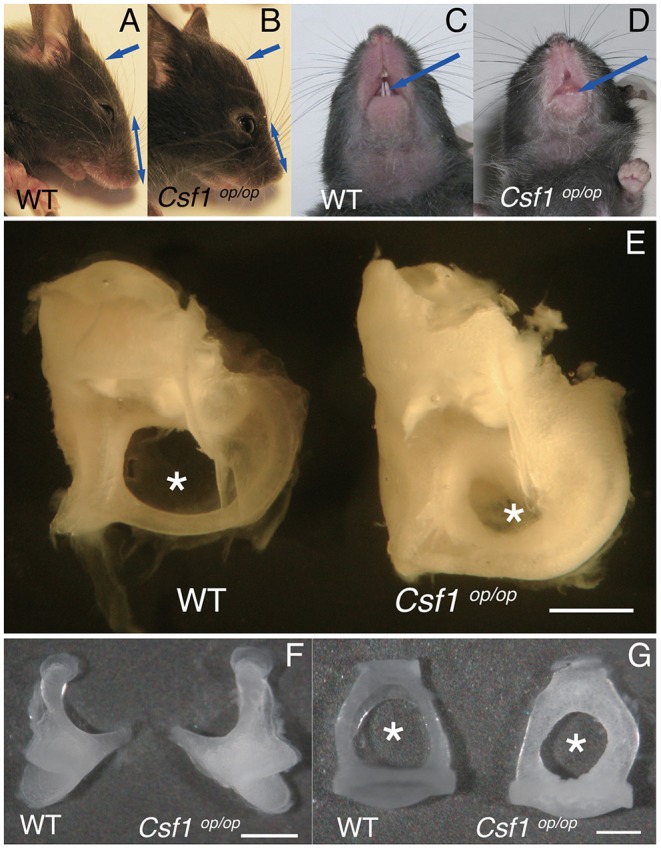
Macroscopic phenotypes of the skull and ear in *Csf1*^*op*/*op*^ mice. **(A–D)** *Csf1*^*op*/*op*^ mice are clearly recognized by a distinctly domed skull and short snout (**A,B**, arrows and double headed arrows) and failure of eruption of the incisors (**C,D**, arrows). **(E)** Image of dissected cochleae from a representative set of *wild type* (*WT*) and *Csf1*^*op*/*op*^ mice. The subarcuate fossa surrounded by the superior semicircular canal is remarkably small due to excessive bone formation of the inner ear in *Csf1*^*op*/*op*^ mice (asterisks). Hypertrophy of the incus **(F)** and stapes **(G)** taken from *Csf1*^*op*/*op*^ mice is shown in comparison to *WT* mice. The obturator foramen between the anterior and posterior crus is also remarkably small (asterisks). Bars: 1 mm for **(E)**, 500 μm for **(F)**, and 200 μm for **(G)**.

*Csf1*^*op*/*op*^ mice could be clearly recognized by approximately 10 days of age by a distinctly domed skull and short snout (Figures 1A,B, arrows and double headed arrows), and by failure of eruption of the incisors (Figures 1C,D, arrows), as previously reported (12). We studied the macroscopic phenotype of the inner ear in *Csf1*^*op*/*op*^ mice. Cleared inner ears from *WT* and *Csf1*^*op*/*op*^ mice demonstrated that the otic capsules in *Csf1*^*op*/*op*^ mice are thick and opaque. In particular, the subarcuate fossa surrounded by the superior semicircular canal showed shrinkage in *Csf1*^*op*/*op*^ mice (Figure 1E, asterisks), reflecting excessive bone formation in the inner ear. Moreover, ossicles including the incus and the stapes demonstrated bone thickening (Figures 1A,B). The obturator foramen of the stapes pierced by the stapedial artery was also smaller in *Csf1*^*op*/*op*^ mice compared with *WT* littermates due to excessive bone formation of the stapes (Figures 1F,G).

### The Otic Capsules Surrounding the Membranous Labyrinth of *Csf1^*op*/*op*^* Mice Demonstrate Bone Thickening and Spongy Bone Degeneration

**Figure 2:**
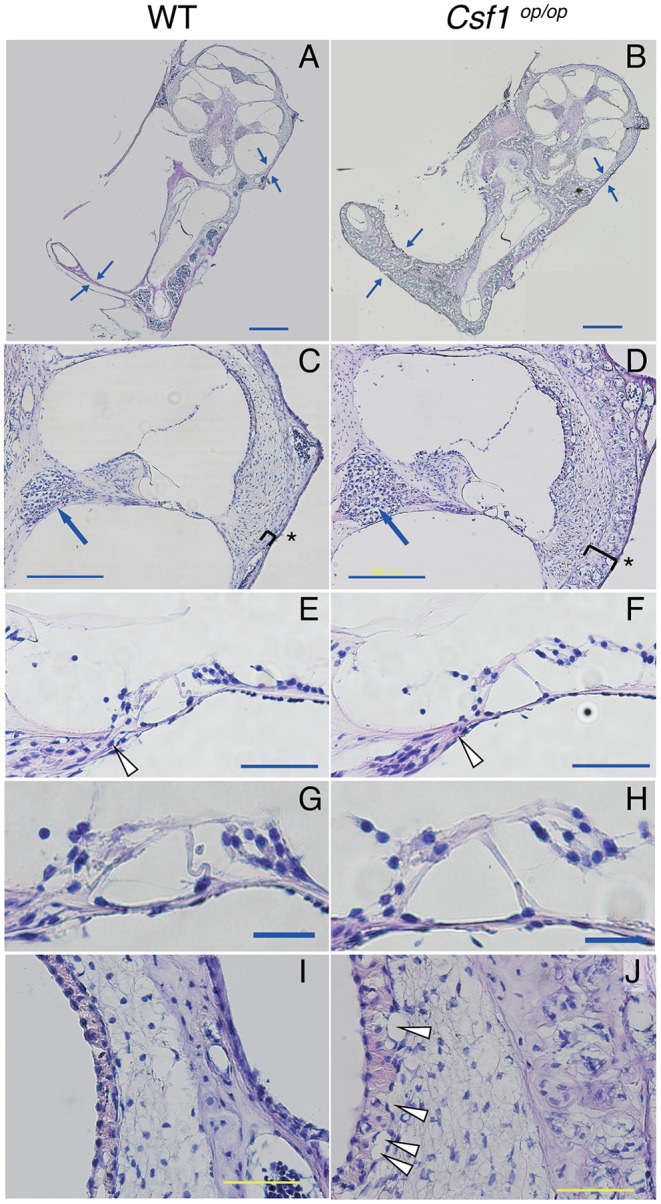
Histological analyses demonstrated that the otic capsules of *Csf1*^*op*/*op*^ mice have bone thickening and spongy bone degeneration. **(A,B)** Irregular lesions in *Csf1*^*op*/*op*^ mice cause bone thickening of the otic capsule with spongy bone degeneration (arrows). Despite thickening of the otic capsule (parentheses), the spiral ganglion (arrows in **C,D**), the habenula perforata (arrowheads in **E,F**), and the organ of Corti **(G,H)** appear normal because at least one row of IHC, three rows of OHCs, and the triangle formed by two pillar cells appear to be preserved, whereas vacuolar formation in the basal cell layer of the stria vascularis was observed in *Csf1*^*op*/*op*^ mice (arrowheads in **I,J**). The organ of Corti in the cochlear basal turn is shown. Bars: 500 μm for **(A,B)**, 200 μm for **(C,D)**, 50 μm for **(E,F,I,J)**, 20 μm for **(G,H)**.

Cryostat sections of the inner ear stained with hematoxylin-eosin showed that the otic capsules surrounding the membranous labyrinth of *Csf1*^*op*/*op*^ mice were thickened and demonstrated spongy bone degeneration (Figures 2A,B arrows, *n* = 4 for each genotype). Higher magnification images of the cochlear duct stained with hematoxylin-eosin also demonstrated excessive spongy bone formation in the lateral wall of the cochlea (Figures 2C,D, brackets and asterisks). The mean thickness of bony wall of the otic capsule was 37.5 ± 3.1 μm in *WT* mice in the basal turn, whereas that in *Csf1*^*op*/*op*^ mice was 76.1 ± 2.3 μm. The difference in thickness of otic capsule was statistically significant (unpaired *t*-test, *p* < 0.001). In contrast, normal formation of Rosenthal's canal was observed, which contains the spiral ganglion in the cochlear modiolus (Figures 2C,D, arrows) and the osseous spiral lamina (Figures 2E,F, arrrowheads). The mean cross-sectional area of Rosenthal's canal in the basal turn was 1.85 ± 0.08 ×10^4^ μm^2^ in *WT* mice while that in *Csf1*^*op*/*op*^ mice was 1.94 ± 0.27 ×10^4^ μm^2^. The difference in the cross-sectional area of Rosenthal's canal was not statistically significant (unpaired *t*-test, *p* = 0.76). In addition, the mean width of habenula perforata in the osseous spiral lamina of *WT* mice was 16.7 ± 1.3 μm, while that in *Csf1*^*op*/*op*^ mice was 21.1 ± 2.0 μm. The difference in the width of habenula perforata was not statistically significant (unpaired *t*-test, *p* = 0.11). The gross morphology of the organ of Corti in the inner ear of *Csf1*^*op*/*op*^ mice also appeared to be intact as one row of inner hair cells, three rows of outer hair cells, and the triangle formed by two pillar cells were at least preserved (Figures 2E–H). These findings are consistent with the development of the spiral ganglion and innervation of the auditory hair cells preceding the formation of the bone surrounding the cochlear duct. Finally, higher magnification images of SV revealed vacuole formation between the basal and intermediate cell layer of SV (Figures 2I,J, arrowheads).

### Four-Week Old *Csf1^*op*/*op*^* Mice Show Significant Elevation of ABR Thresholds Compared With *WT* Littermates

**Figure 3:**
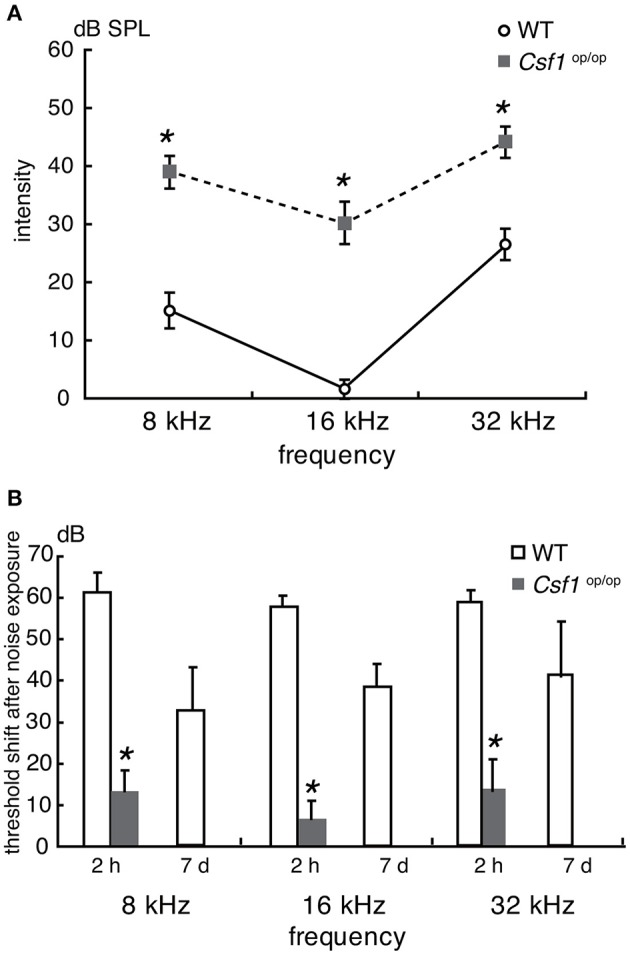
Auditory brainstem response analyses revealed that *Csf1*^*op*/*op*^ mice have mild to moderate hearing loss. **(A)** Auditory brainstem response (ABR) thresholds (decibels of sound pressure level, dB SPL) were measured in 4-week-old *Csf1*^*op*/*op*^ mice and *wild type* (*WT*) littermates. Male and female mice were analyzed together. *Csf1*^*op*/*op*^ mice show a higher ABR threshold at 8, 16, and 32 kHz, indicating that *Csf1*^*op*/*op*^ mice have mild to moderate hearing loss. **(B)** ABR thresholds were also measured 2 h and 7 days after noise exposure to 8 kHz octave band noise at 120 dB SPL for 1 h. *Csf1*^*op*/*op*^ mice are less susceptible to acoustic overstimulation, and the difference in the threshold shift in ABR 2 h after noise exposure compared with *WT* mice is statistically significant. Bars: mean ± SE. *p < 0.05.

To assess the auditory function of *Csf1*^*op*/*op*^ mice, ABR thresholds in response to tone burst stimuli at 8, 16, and 32 kHz were measured in 4-week old *Csf1*^*op*/*op*^ mice and *WT* littermates (*n* = 4 for each genotype). Thresholds in *WT* mice were 15.0 ± 2.9, 1.3 ± 1.3, and 26.3 ± 2.4 dB SPL at 8, 16, and 32 kHz, respectively, whereas thresholds in *Csf1*^*op*/*op*^ mice were 38.8 ± 2.4, 30.0 ± 3.5, and 43.8 ± 2.4 dB SPL at 8, 16, and 32 kHz, respectively. Thresholds for all frequencies measured were significantly higher in *Csf1*^*op*/*op*^ than in *WT* mice (Figure 3A), indicating that *Csf1*^*op*/*op*^ mice show moderate hearing loss. When animals were exposed to 8 kHz octave band noise at 120 dB SPL for 1 h, the threshold shifts in *WT* mice were 61.3 ± 2.4, 66.3 ± 1.3, 58.8 ± 1.3 dB at 8, 16, and 32 kHz, respectively, while the threshold shifts in *Csf1*^*op*/*op*^ mice were 13.8 ± 2.4, 7.5 ± 2.5, 13.8 ± 3.8 dB at 8, 16, and 32 kHz, respectively (Figure 3B). In addition to threshold shifts after noise exposure, raw data of ABR thresholds for all frequencies 2 h after noise exposure were significantly lower in *Csf1*^*op*/*op*^ mice than in *WT* mice. Moreover, exposure to the 8 kHz octave band noise at 120 dB SPL for 1 h caused permanent threshold shifts in *WT* mice, whereas *Csf1*^*op*/*op*^ mice showed only transient threshold shifts and recovery at 7 days after noise exposure to the same levels before noise exposure. Third, in *Csf1*^*op*/*op*^ mice, the mean latencies of the wave I and the I-III interwave intervals at 8 kHz, 100 dB SPL were 2.74 ± 0.011 and 2.14 ± 0.016, and the mean latencies of the wave I and the I-III interwave intervals at 8 kHz, 70 dB SPL were 2.91 ± 0.028 and 2.19 ± 0.022, respectively. In *WT* mice, the mean latencies of the wave I and the I-III interwave intervals at 8 kHz, 70 dB SPL were 2.71 ± 0.016 and 2.13 ± 0.15, and the mean latencies of the wave I and the I-III interwave intervals at 8 kHz, 40 dB SPL were 2.92 ± 0.021 and 2.18 ± 0.15, respectively. The latencies of waves I and III were consistently delayed in *Csf1*^*op*/*op*^ mice without any lengthening of interval between waves I and III, potentially suggesting conductive hearing loss in *Csf1*^*op*/*op*^ mice.

Taken together, these findings suggest that moderate hearing loss shown in *Csf1*^*op*/*op*^ mice was due to, at least in part, deformity of the ossicles and the otic capsule leading to a conductive hearing disorder, although the possibility of mixed hearing loss cannot be completely excluded.

### *Csf1^*op*/*op*^* Mice Demonstrate a Significant Deficiency in the Number of Resident Macrophages in the Spiral Ligament and Stria Vascularis

**Figure 4:**
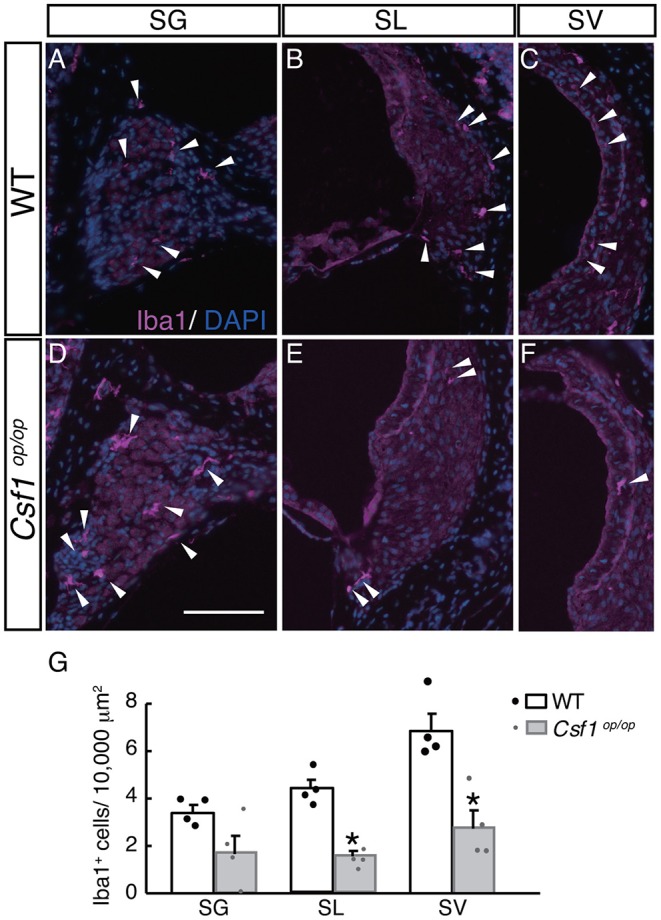
Distribution and density of Iba1-positive macrophages in each part of the cochlea of *wild type* and *Csf1*^*op*/*op*^ mice. Iba1-positive macrophages are found in the spiral ganglion **(A,D)**, spiral ligament **(B,E)**, and intermediate cell layer of the stria vascularis **(C,F)** in both *wild type* (*WT*) and *Csf1*^*op*/*op*^ mice at the age of 4 weeks. While there is no statistically significant difference in the density of Iba1-positive macrophages in the spiral ganglion, a significant reduction in the density of Iba1-positive cells is observed in *Csf1*^*op*/*op*^ mice, suggesting Csf1 dependency of resident macrophages in the spiral ligament and stria vascularis (**G**, single plots are also shown; *n* = 4 for each genotype). Bar: 200 μm for **(A–F)** (shown only in **D**). *p < 0.05.

To examine the roles of Csf1 signaling in the maintenance and distribution of resident macrophages in the mouse cochlea, the density of resident macrophages was examined with immunohistochemistry for Iba1 on the mid-modiolar section of 4-week old *WT* and *Csf1*^*op*/*op*^ mice. Non-sensory parts of the cochlea including the SG, SL, and SV were densely populated with resident macrophages identified by Iba1 immunostaining, as previously shown (3) (Figures 4A–C). These cells had a characteristic morphology with several ramified processes that branched from the main axis of the cell body. However, the density of the cochlear macrophage population labeled with Iba1 was significantly decreased in *Csf1*^*op*/*op*^ mice except in the SG (Figures 4D–F). The numbers of Iba1-positive cells per 10,000 μm^2^ in the cochlea of *WT* mice were 3.42 ± 0.29, 4.35 ± 0.36, and 6.83 ± 0.69 in the SG, SL, and SV, respectively, whereas in *Csf1*^*op*/*op*^ mice these numbers were 1.71 ± 0.71, 1.55 ± 0.17, 2.77 ± 0.71 in the SG, SL, and SV, respectively. The difference in density of Iba1-positive cells between *WT* and *Csf1*^*op*/*op*^ mice was statistically significant in both SL and SV (Figure 4G, *n* = 4 for each genotype).

Taken together, Csf1 deficiency in the cochlea of 4-week old mice causes a significant decrease in the number of resident macrophages expressing Iba1, indicating that Csf1 signaling is required for the maintenance of cochlear resident macrophages in the SL and SV.

## Discussion

In this study, we examined the phenotype of the inner ear in *Csf1*^*op*/*op*^ mice. The ossicles of *Csf1*^*op*/*op*^ mice macroscopically showed bone thickening, and the otic capsules of *Csf1*^*op*/*op*^ mice were also thick and opaque. Histological analyses demonstrated that the otic capsules of *Csf1*^*op*/*op*^ mice were thickened and showed spongy bone degeneration. ABR measurements revealed significant elevation of thresholds in 4-week old *Csf1*^*op*/*op*^ mice compared with their *WT* littermates, indicating that *Csf1*^*op*/*op*^ mice demonstrate hearing loss due to, at least in part, deformity of the ossicles and bone capsule of the inner ear. Furthermore, *Csf1*^*op*/*op*^ mice possess a significant deficiency in the number of resident macrophages in the spiral ligament and stria vascularis, but not in the spiral ganglion. These data provide evidence that Csf1 signaling is important not only for bone formation in the inner ear, but also for the maintenance of resident macrophages in the spiral ligament and stria vascularis of the adult mouse cochlea.

Interactions between the organ of Corti and the structures of the cochlea, such as the otic capsule and ossicles, are essential for hearing. In the embryo, the cochleae are formed through bidirectional signaling between the sensorineural structures and developing bone (14). Young *Csf1*^*op*/*op*^ mice have excessive accumulation of bone without marrow cavities, increases in bone matrix formation (12), and, as demonstrated by our findings, spongy degeneration in the otic capsule that is associated with hearing impairment observed in ABR measurements. These observations show that *Csf1*^*op*/*op*^ mice have a restricted capacity for bone remodeling, with severely reduced numbers of osteoclasts in the bone (15). *Csf1*^*op*/*op*^ mice are used as a model for human hearing impairment observed in patients with osteopetrosis. Dozier et al. reported a case series of 32 patients with autosomal recessive osteopetrosis, which demonstrated that 26% of infants' ears showed hearing loss during the first year of life, and a total of 78% of children's ears demonstrated hearing loss during the study period. Of the children's ears with hearing loss, 100% had a conductive component and 26% had an additional sensorineural component (mixed hearing loss); cochleovestibular nerve conduction was normal in 100% of infants and 78% of children (16). While there are cases of osteopetrosis causing sensorineural hearing loss in humans, it has been hypothesized that this is due to auditory nerve compression from bony hypertrophy. In such cases, there are also reports that cochlear implants have been useful in patients with osteopetrosis who have deafness (17). Although osteopetrosis is genetically diverse, mutations in *CSF1* have been reported as autosomal recessive in humans (18). In the present study, *Csf1*^*op*/*op*^ mice showed no nerve compression; therefore, hearing loss is thought to be largely due to conductive components such as spongy dysplasia of bone tissue in the ossicles and otic capsule. Our findings that *Csf1*^*op*/*op*^ mice showed reduced elevation of ABR thresholds and quick recovery after acoustic overstimulation corroborates the presence of conductive hearing loss in *Csf1*^*op*/*op*^ mice, although the possibility of mixed hearing loss cannot be completely excluded. Because *Csf1*^*op*/*op*^ mice were analyzed at the age of 4 weeks in the present study, cochlear bony structures in *Csf1*^*op*/*op*^ mice may change with age particularly with regard to nerve compression in the cochlear modiolus or osseous spiral lamina. In addition, recent studies on resident macrophages in the stria vascularis revealed specific roles as perivascular resident macrophage-like melanocytes (19, 20).

Csf1 is the most potent growth factor for macrophage differentiation (21, 22). In *Csf1*^*op*/*op*^ mice, the production of functional M-CSF protein is impaired because of a defect in the coding region of the *Csf1* gene (15, 23). In *Csf1*^*op*/*op*^ mice, the numbers of mature macrophages are reduced in many tissues, including the liver, spleen, lungs, and brain, because Csf1 deficiency results in widespread defects in monocyte/macrophage differentiation and osteoclast development. The failure of osteoclast development and differentiation in *Csf1*^*op*/*op*^ mice results in impaired bone resorption and remodeling, leading to systemic osteopetrosis (15, 24–26). In our study, the otic capsules of *Csf1*^*op*/*op*^ mice have histological changes in the inner ear, including bone thickening and spongy bone degeneration, which is compatible with previous studies (15, 24–26). In addition, *Csf1*^*op*/*op*^ mice possess a significant deficiency in the number of resident macrophages in the spiral ligament and stria vascularis, but not in the spiral ganglion.

There are several reasons why *Csf1*^*op*/*op*^ mice did not show complete loss of cochlear resident macrophages. Maternal Csf1, mainly produced from the uterus, is thought to support early development of *Csf1*^*op*/*op*^ fetuses, which develop in the absence of Csf1 after birth. Milk-derived Csf1 does not seem to play a role in the development of macrophages in *Csf1*^*op*/*op*^ mice. Accordingly, postnatal changes in macrophage subpopulations reflect a loss of Csf1 function (27). Another consideration is compensation of Csf1 signaling by IL-34. Most tissue macrophage populations are markedly reduced, but splenic macrophages are only slightly affected in *Csf1*^*op*/*op*^ mice (28, 29), whereas *Il-34* null mice display a normal population of microglia and Langerhans cells (30). However, recent study revealed distinct requirement for Csf1 and IL-34 in the development and maintenance of microglia population (31), therefore further investigation should be required for elucidation of the detailed mechanisms of Csf1 signaling in the development of cochlear resident macrophages. According to previous studies in *Csf1*^*op*/*op*^ mice and *Il-34* null mice, tissue resident macrophages are generally divided into two groups: Csf1-dependent macrophages and IL-34-dependent macrophages (32). Therefore, based on our results, the majority of resident macrophages in the spiral ligament and stria vascularis are Csf1-dependent, while the population of resident macrophages in the spiral ganglion is relatively Csf1-independent. Moreover, there is a possibility that a heterogeneous population of macrophages resides in each part of the mouse inner ear. Further studies are required to elucidate the characteristics or mechanisms of differentiation of subtypes of cochlear resident macrophages.

In conclusion, our findings reveal unique roles for Csf1 signaling in bone morphogenesis and maintenance of resident macrophages in the mouse inner ear. These mechanisms could serve as a novel pharmacological target to treat the skeletal or hearing manifestations of osteopetrosis and other skeletal diseases.

## Data Availability Statement

The datasets generated for this study are available on request to the corresponding author.

## Ethics Statement

This study was carried out in accordance with the recommendations of the NIH Guide for the Care and Use of Laboratory Animals. The protocol was approved by the Animal Research Committee of Kyoto University Graduate School of Medicine (No. 170510, MedKyo18117).

## Author Contributions

TO conceived and designed the experiments, performed the experiments, analyzed the data, wrote the paper. IK analyzed the results and prepared the manuscript.

### Conflict of Interest

The authors declare that the research was conducted in the absence of any commercial or financial relationships that could be construed as a potential conflict of interest.
